# Supplementary material for: Fatiguing effects of indirect vibration stimulation in upper limb muscles: pre, post and during isometric contractions superimposed on upper limb vibration
Source: R Soc Open Sci. 2019 Oct 2;6(10):190019. doi: 10.1098/rsos.190019 (PMC6837201; doi:10.1098/rsos.190019)
Supplement: Figures 14 to 25, Normalised mean EMGrms values for the Biceps, Triceps and the Forearm [file rsos190019supp1.docx]

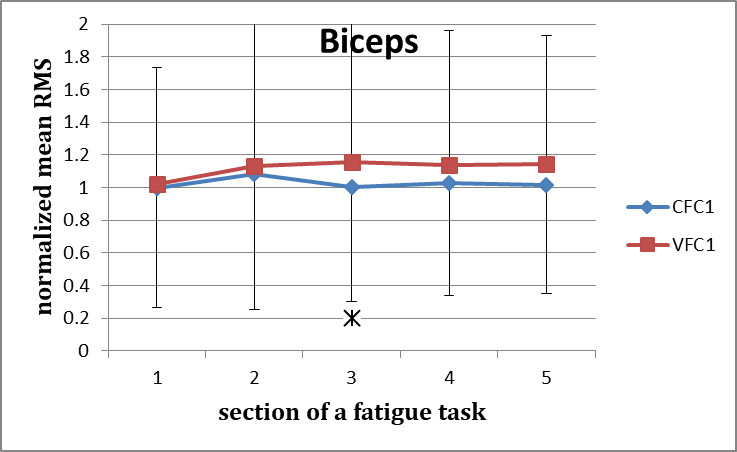

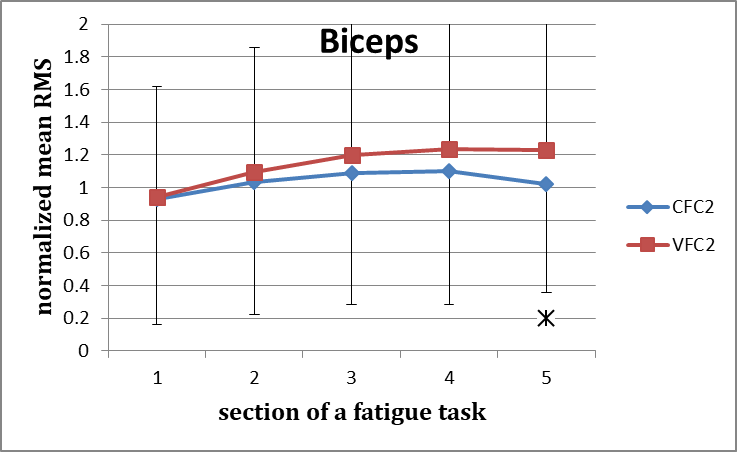
Figure 14 Figure 15
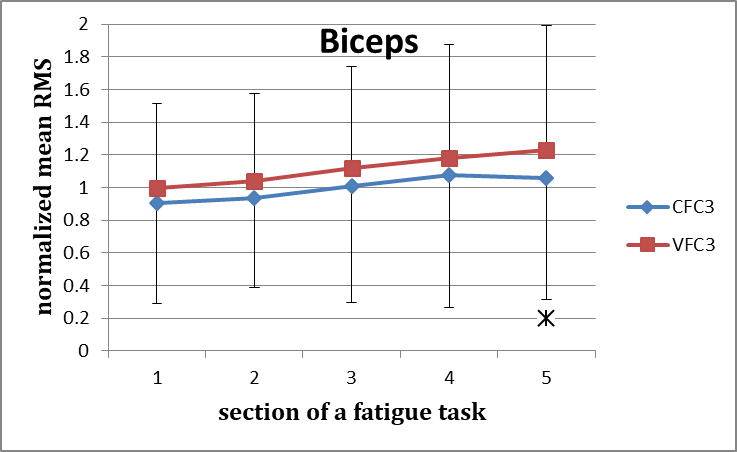

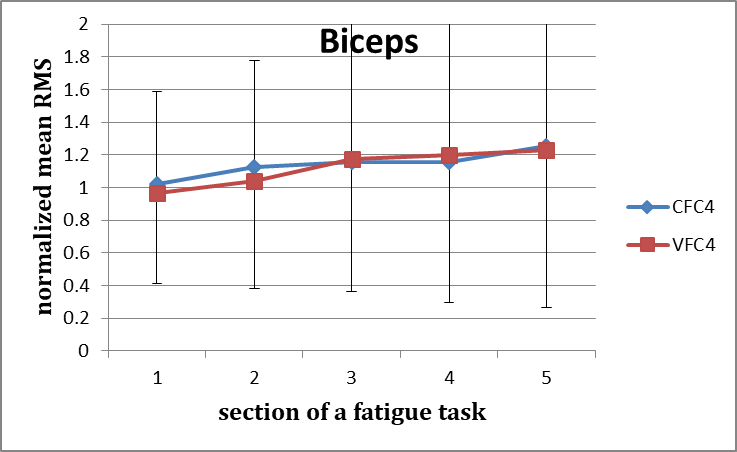
Figure 16 Figure 17

Figures 14-17: Normalised mean EMGrms values for the Biceps for the five consecutive sections of the fatigue effort during the progression of each of the successive four fatiguing exercise efforts performed, under control and vibration condition. Effort 1- CFC1 vs VCF1 (Figure 14), Effort 2- CFC2 vs VCF2 (Figure 15), Effort 3- CFC3 vs VCF3 (Figure 16), and Effort 4- CFC4 vs VCF4 (Figure 17).


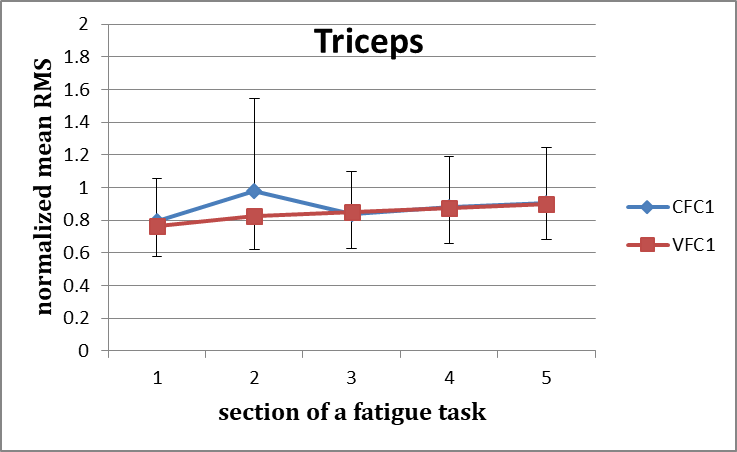

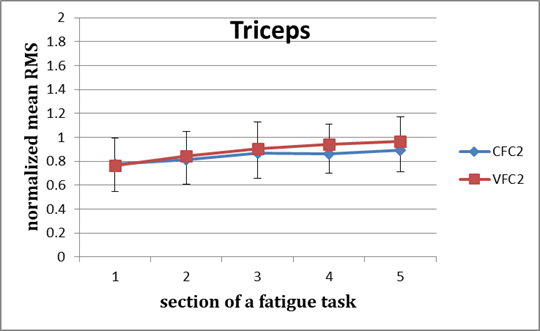
Figure 18 Figure 19
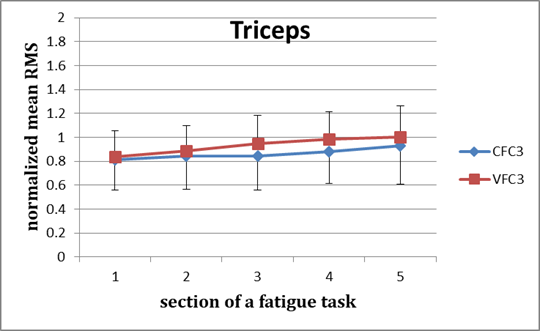

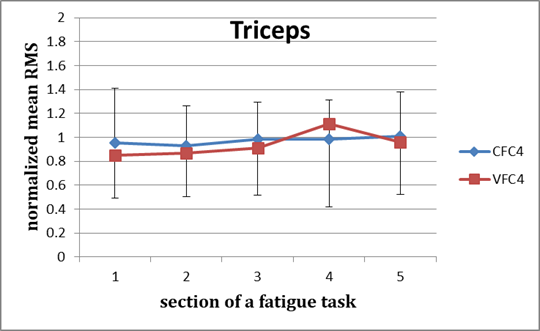
Figure 20 Figure 21

Figures 18-21: Normalised mean EMGrms values for the Triceps for the five consecutive sections of the fatigue effort during the progression of each of the successive four fatiguing exercise efforts performed, under control and vibration condition. Effort 1- CFC1 vs VCF1 (Figure 18), Effort 2- CFC2 vs VCF2 (Figure 19), Effort 3- CFC3 vs VCF3 (Figure 20), and Effort 4- CFC4 vs VCF4 (Figure 21).


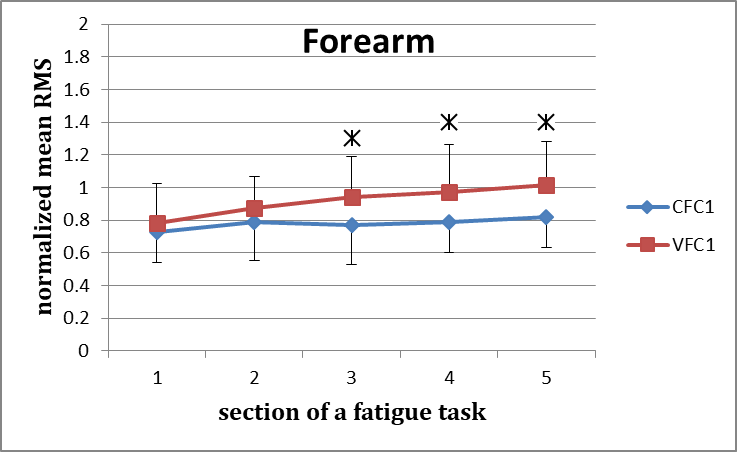

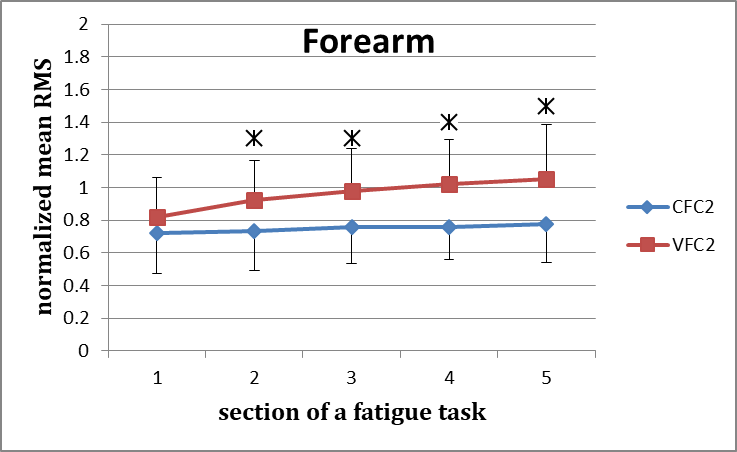
Figure 22 Figure 23
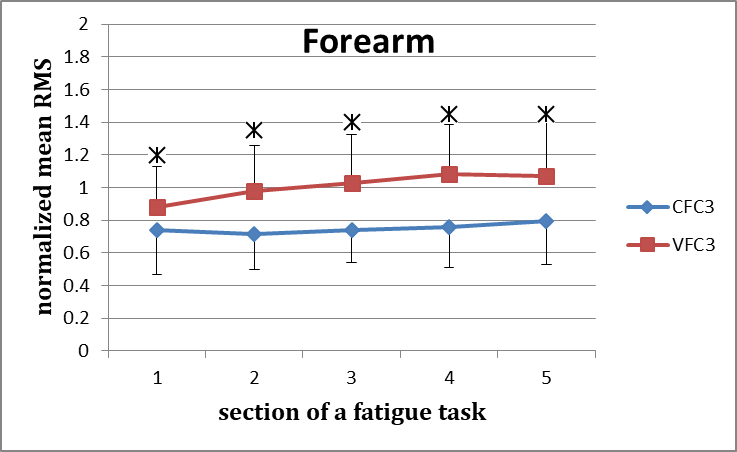

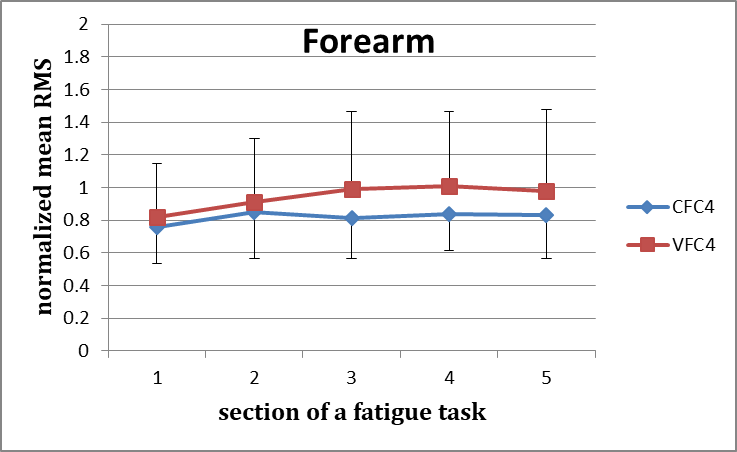
Figure 24 Figure 25

Figures 22-25: Normalised mean EMGrms values for the Forearm for the five consecutive sections of the fatigue effort during the progression of each of the successive four fatiguing exercise efforts performed, under control and vibration condition. Effort 1- CFC1 vs VCF1 (Figure 22), Effort 2- CFC2 vs VCF2 (Figure 23), Effort 3- CFC3 vs VCF3 (Figure 24), and Effort 4- CFC4 vs VCF4 (Figure 25).
